# Results of Onlay Preputial Flap Urethroplasty for the Single-Stage Repair of Mid- and Proximal Hypospadias

**DOI:** 10.3389/fped.2018.00019

**Published:** 2018-02-08

**Authors:** Ricardo González, Anja Lingnau, Barbara Magda Ludwikowski

**Affiliations:** ^1^Pediatric Surgery and Urology, Kinder- und Jugendkrankenhaus AUF DER BULT, Hanover, Germany; ^2^Department of Urology, Charité Universitätsmedizin Berlin, Berlin, Germany

**Keywords:** hypospadias, preputial flap, one-stage repair, uroflow, onlay urethroplasty, double face flap

## Abstract

**Aims:**

To report current results of preputial flap onlay urethroplasty using the principle of the total preputial flap (TPF) for the one-stage repair of mid- and proximal hypospadias.

**Methods:**

This study was a retrospective chart review of patients in a prospectively kept database of all hypospadias operations performed at two institutions from January 1 2011 to August 2017. Inclusion criteria: all patients who underwent hypospadias repair using a preputial only flap urethroplasty based on the principle of the TPF. Demographic data, duration of follow-up, complications, and reoperations were recorded. A successful result was considered to be a straight penis, a glanular meatus, and absence of voiding symptoms. Whenever possible an uroflow was obtained during the follow-up visits.

**Results:**

Forty-nine children met the inclusion criteria. All patients had marked penile curvature. Three patients had chromosomal abnormalities. The mean age at the time of surgery was 22 months (11–110) and the mean duration of follow-up 23.4 months (1–79). In 48 cases, the urethral plate could be preserved without dividing it. The penile curvature was corrected with chordectomy alone in 10 patients, 38 required a dorsal plication of the tunica albuginea, and 1 required an additional ventral dermal graft. In 38 patients (77.5%), the initial operation was successful, and no further operations were needed. There were eight urethrocutaneous fistulas, three dehiscences of the glans approximation. One patient suffered a wound infection and partial loss of the flap.

**Conclusion:**

One-stage repair of mid- and proximal hypospadias preserving the urethral plate and using a TPF for the urethroplasty and coverage of the ventral penis is successful in 77.5% of cases. Complications in the remaining patients were easily managed or did not require treatment. Compared to a planned two-stage approach, the technique described in this report resulted in significantly fewer procedures till complete resolution of the problem.

## Introduction

The surgical repair of hypospadias involving an urethroplasty and repair of a ventral curvature greater than 20° (1, 2) after releasing the ventral penile skin is often referred to as repair of middle and proximal or posterior hypospadias (3). The correction of these malformations continues to challenge surgeons, and there is no agreement as to the best technique for its correction. As opposed to repair of distal hypospadias without penile curvature which is mainly cosmetic (4), the repair of mid- and proximal hypospadias has also functional implications. Over decades, trends have swung between staged repairs (5–7) to one-stage repair (8–11). At present, the prevailing tendency favors the use of stage repair advocated by Bracka et al. (12–14).

Nevertheless, based on our previous experience and given the fact that some literature reports equivalent results with various techniques, we have continued to use the technique described by one of us more than 20 years ago (15), which consists of avoiding the division of the urethral plate in the majority of the cases and reconstruction of the urethra with an onlay flap derived from the outer layer of a total preputial flap (TPF) and when the ventral skin deficiency required it, a double face preputial flap was used (16). Onlay urethroplasty for the repair of hypospadias has yielded good long-term results (10, 17). Here, we report our experience with the abovementioned technique in consecutive cases operated by two surgeons (Barbara Magda Ludwikowski and Anja Lingnau).

## Materials and Methods

This study was a retrospective review of patient’s records operated at two institutions. In institution A, a prospective database of all hypospadias operations was kept since 2011. At institution B, records were located from an electronic database search for onlay urethroplasty and hypospadias repair. Ethical approval for this review was obtained at institution A and not required at institution B.

Inclusion criteria: all patients who underwent hypospadias repair using a preputial only flap urethroplasty. When the ventral skin deficiency required it, a double face preputial flap was used. All flaps were prepared using the TPF technique and were transferred ventrally by creating a buttonhole in its pedicle to avoid penile rotation (16) Exclusion criteria: previous circumcision or operation for correction of hypospadias. Demographic data, duration of follow-up, complications, and reoperations were recorded. A successful result was considered to be a straight penis, a glanular meatus, and absence of voiding symptoms. Whenever possible an uroflow was obtained during the follow-up visits.

When the width of the glans was deemed inadequate for a one-stage procedure, preoperative testosterone was administered, and if the response was adequate the operation was carried out within 4 weeks of the last administration.

### Surgical Technique

**Figure 1:**
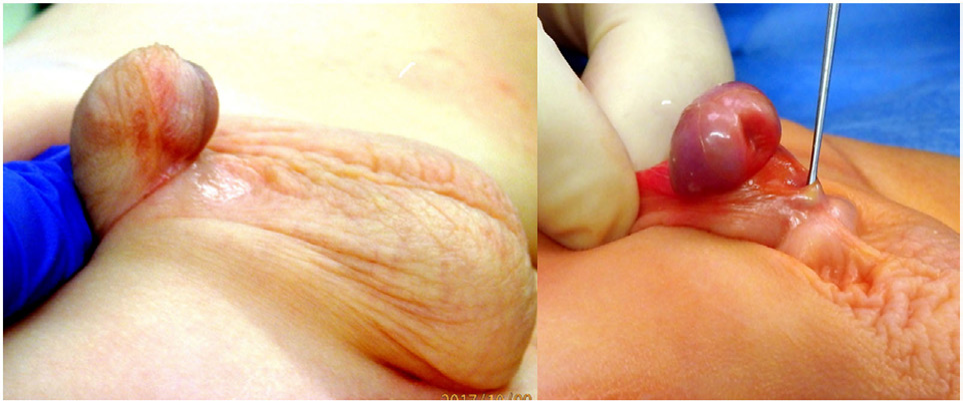
Penoscrotal hypospadias.

**Figure 2:**
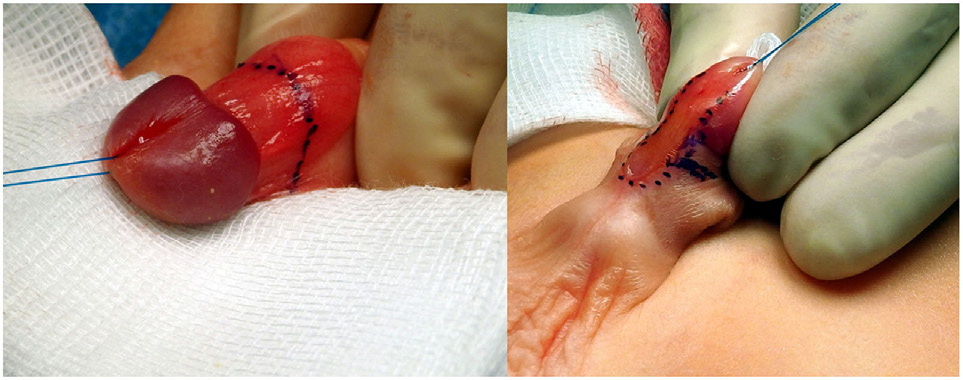
Outline of initial incision.

**Figure 3:**
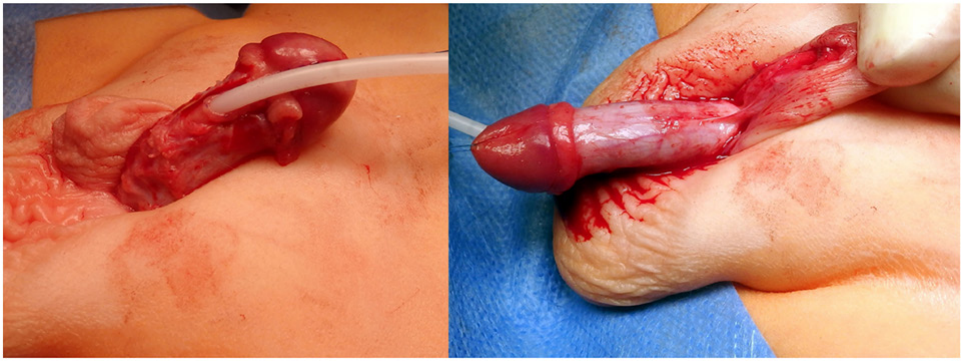
Degloving of penile skin and removal of all fibrous tissues adjacent to the urethral plate and penile urethra.

**Figure 4:**
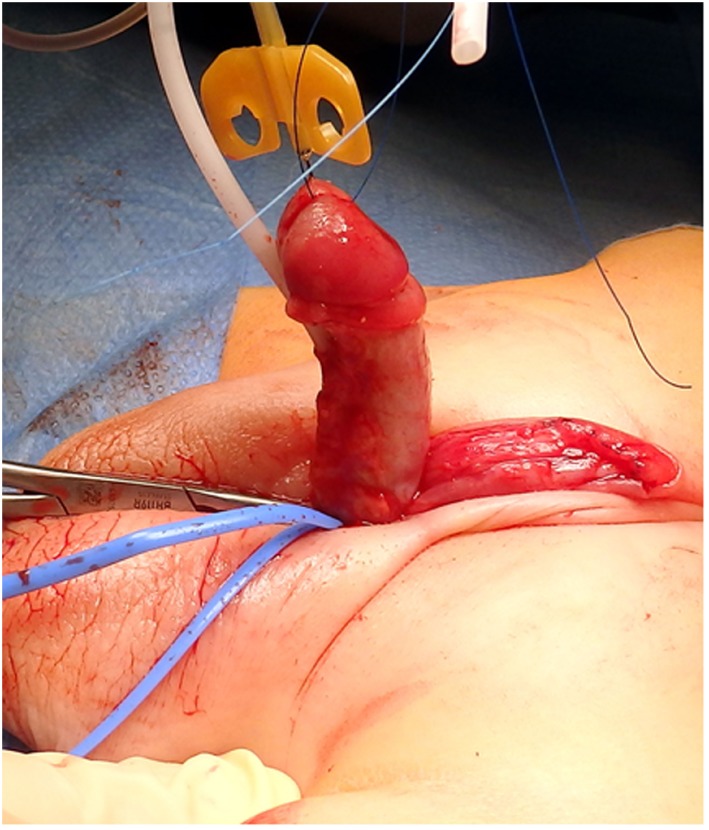
Artificial erection showing residual ventral curvature of about 30°.

**Figure 5:**
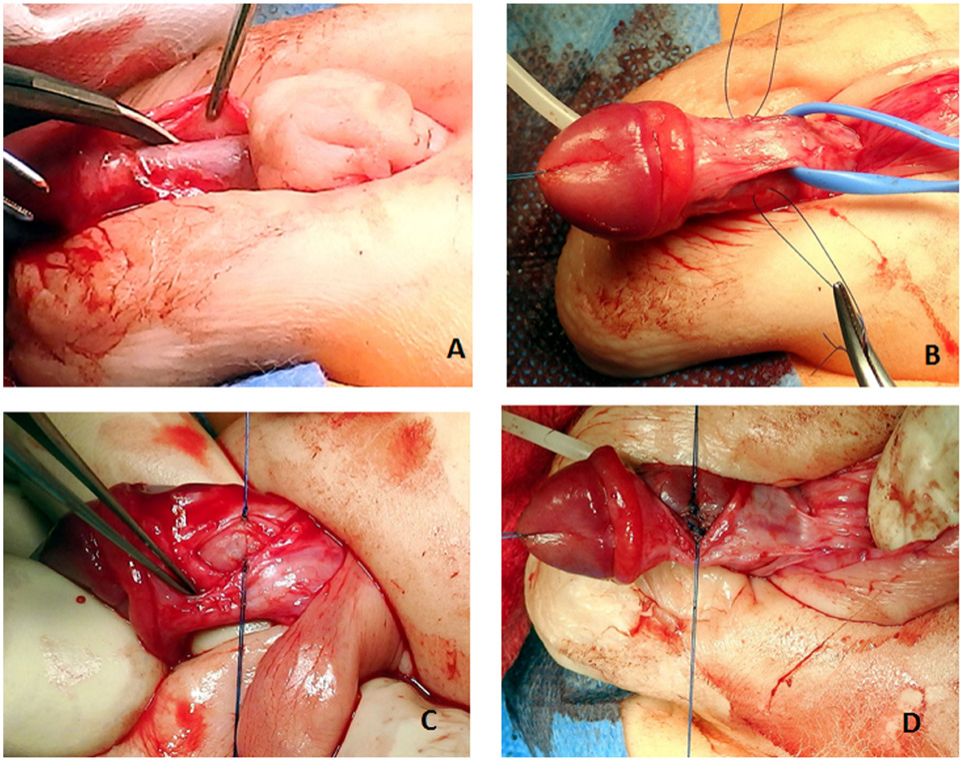
**(A)** Correct plane of dissection to elevate the dorsal neurovascular bundle. **(B)** The bundle is elevated. Two holding sutures are placed laterally at the point of the maximum curvature. **(C)** A midline longitudinal incision is made on the tunica albuginea. **(D)** The incision is closed transversally (Heineke–Mikulicz principle) with 5 or 6-0 non-absorbable monofilament sutures.

**Figure 6:**
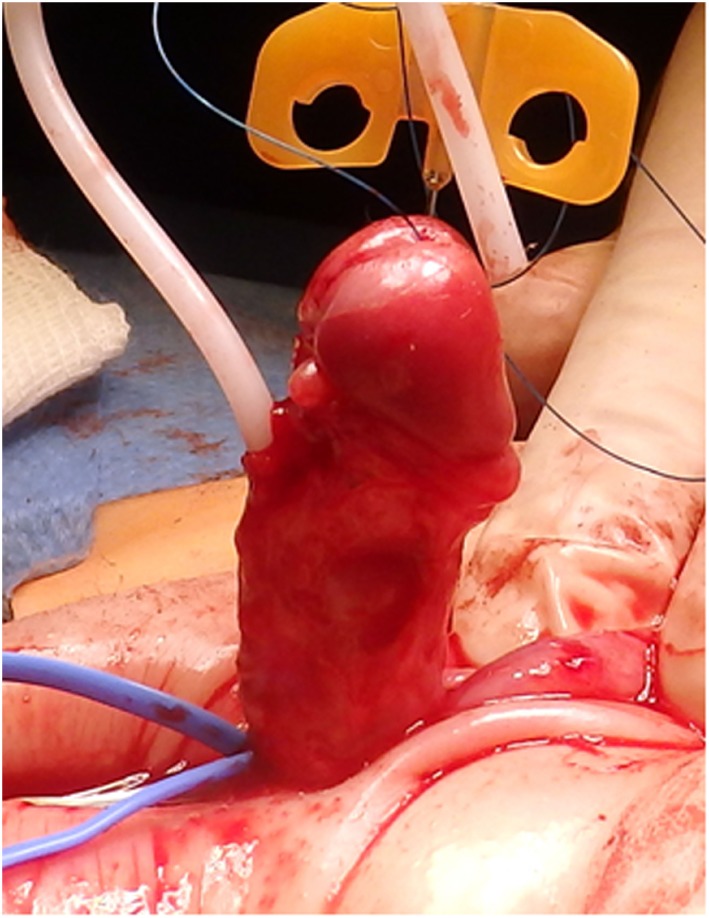
Artificial erection shows complete correction of the curvature.

We illustrate a case of penoscrotal meatus (Figure 1). The operation starts with an incision parallel and 5 mm proximal to the coronal sulcus dorsally that continues ventrally until it meets the lateral boundaries of the urethral plate. The incision is extended proximally along the sides of the urethral plate and surrounds the urethral meatus (Figure 2). The penile skin is degloved along Buck’s fascia, and the fibrotic paraurethral tissue is excised (Figure 3). An artificial erection is performed injecting normal saline into a corpus cavernosus through a fine needle inserted through the glans (18) (Figure 4). If residual curvature remains, the dorsal neurovascular bundle is lifted and a midline incision is made dorsally in the tunica albuginea. The length of the incision varies between 3 and 6 mm according to the severity of the curvature. The incision is closed transversally (Heineke–Mikulicz principle) (19) (Figure 5). If the curvature is very pronounced, the urethral plate is dissected off the corpora cavernosa as described by Mollard and Castagnola (17). Proximal mobilization of the urethra and corpus spongiosum to the bulbous urethra is occasionally necessary to gain urethral length. If after this maneuver the curvature persists, a transverse incision is made in the ventral aspect of the tunica albuginea and covered with a graft of tunica vaginalis or dermis. After correction of the curvature, the artificial erection is repeated (Figure 6).

**Figure 7:**
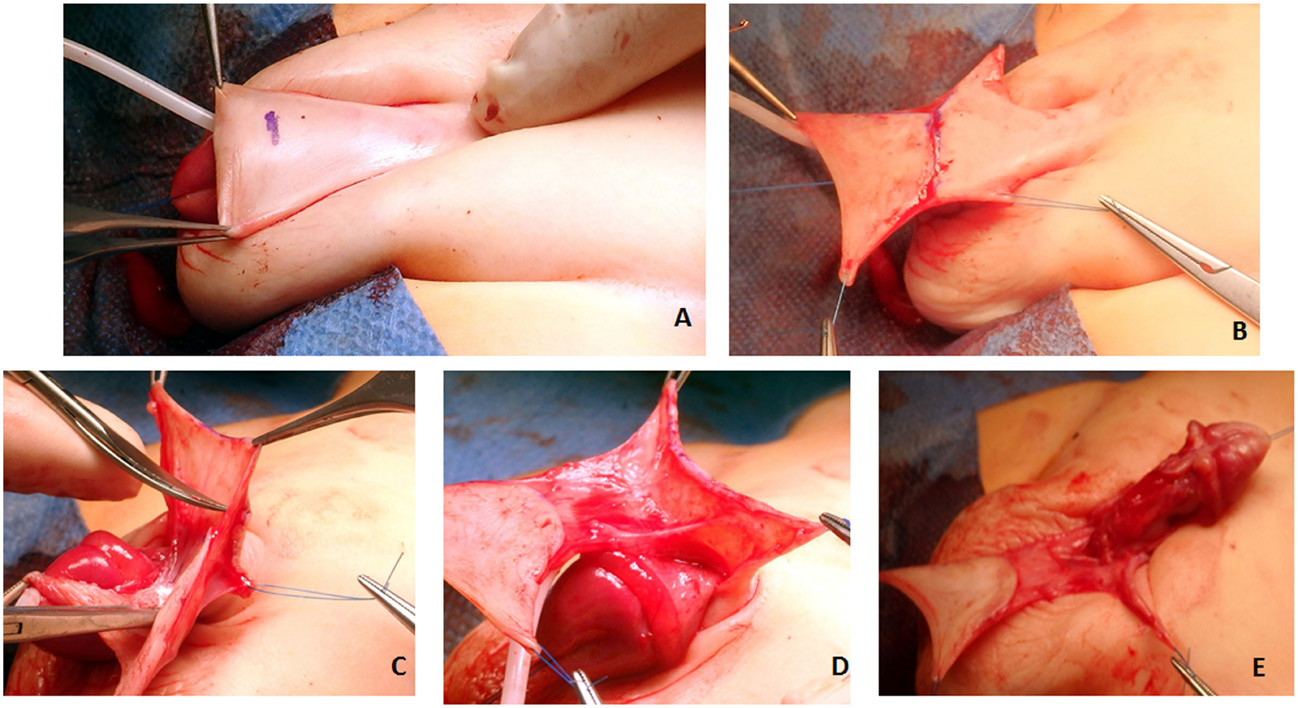
**(A)** The junction of the dorsal shaft skin and the prepuce is marked. **(B)** A superficial incision through skin only is made to start the preparation of the total preputial flap (TPF). Care is taken not to cut the subcutaneous layer containing the blood supply. **(C,D)** Separation of the preputial flap vascular pedicle from the dorsal skin. When in the correct plane, there should be no bleeding. The use of mono or bipolar cautery is strictly avoided in this stage of the operation. **(E)** A puncture hole is made in the midline at the base of the vascular pedicle, and the TPF is transposed to the ventrum of the penis.

**Figure 8:**
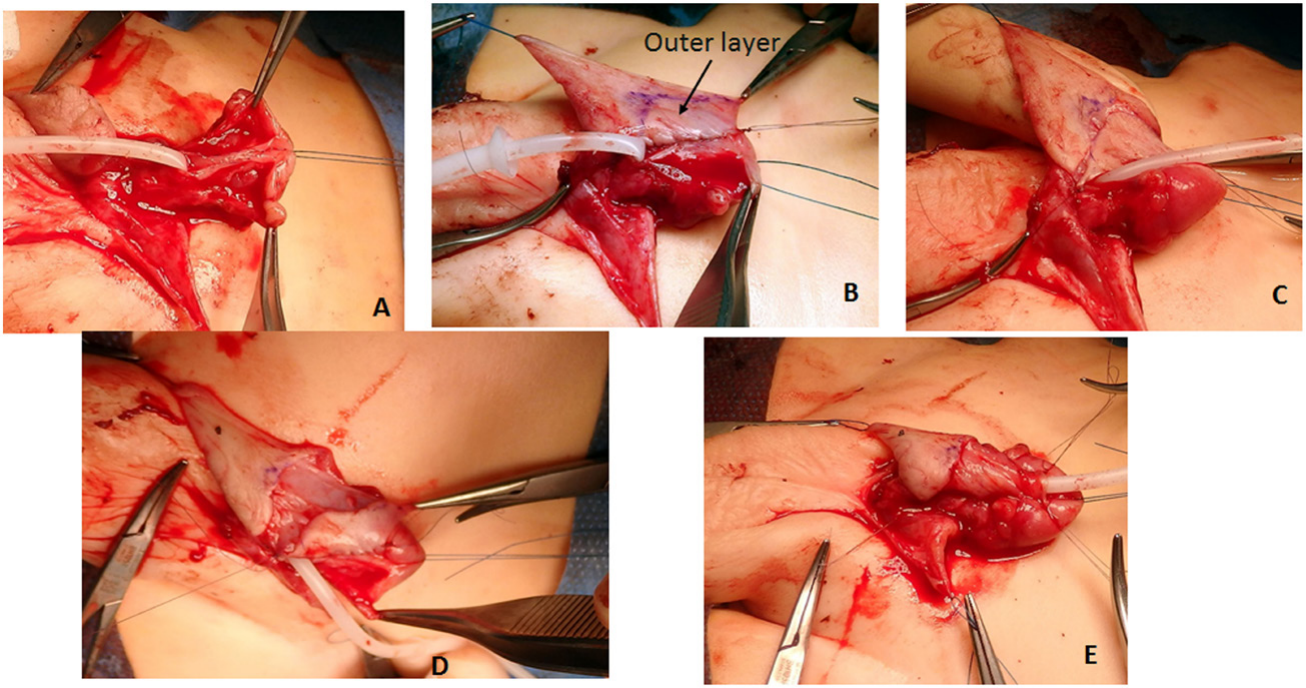
**(A)** Glans wings are prepared and the urethral plate through the glans is clearly delineated. Again here, when in the correct plane of dissection bleeding is minimal. We avoid the use of epinephrine or tourniquet. **(B)** The total preputial flap (TPF) is rotated 90° (clockwise in this case), and the free end of the outer skin is sutured to one side of the urethral plate with a continuous suture of 6-0 poliglecaprone 25. **(C)** A superficial incision is marked outlining the segment of the TPF to be used for the onlay. **(D)** The width of the urethral plate plus onlay should be 12–14 mm. **(E)** The free edge of the onlay flap is sutured to the free edge of the urethral plate with an extra epithelial continuous suture of the same material. The meatus is constructed with 6-0 interrupted sutures; the glans wings are approximated with two deep 5-0 sutures, and the glans skin is closed with 6-0 material.

A TPF is now prepared as previously described (16). The flap is transposed ventrally and rotated 90° (Figure 7). The urethral plate in the glans is outlined with parallel incisions, and the glans is mobilized laterally to allow closure of the glans over the reconstructed urethra. The free edge of outer layer of the preputial l flap is sutured to a side of the urethral plate with a running suture of 6-0 poliglecarpone 25 (Monocryl 25. *Johnson & Johnson Medical GmbH. Ethicon Deutschland, Robert-Koch-Straße 1,D-22851 Norderstedt, Germany*). The planned total width of the urethra (plate and portion of the flap) is outlined, and an incision is made through the skin only of the flap leaving the pedicle intact (Figure 8). This new free edge of the flap is sutured to the other border of the urethral plate with an extraepithelial running suture of 6-0 poliglecarpone 25. When the ventral skin deficiency required it, a double face preputial flap was used. An 8 CH silicone stent is left in the urethra with the proximal end in the bladder. The distal portion of the flap used for the urethroplasty is trimmed as necessary, and the new urethral meatus is reconstructed using interrupted 6-0 sutures. The glans is approximated with two sutures of 5-0 poliglecarpone 25 l, and the skin of the glans is closed with 6-0 sutures (Figure 8).

**Figure 9:**
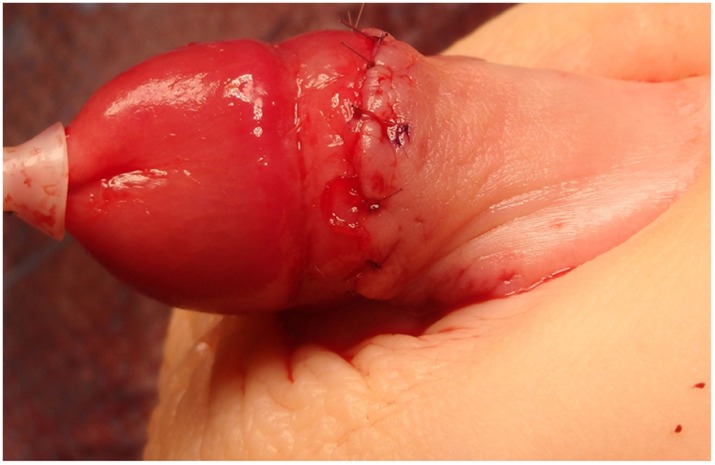
The free edge of the dorsal shaft skin is sutured to the dorsal coronal skin.

Dorsally, the free edge of the penile shaft skin is sutured to the dorsal corona (Figure 9). Attention is now directed at covering the ventral penile skin defect. If there is sufficient penile shaft skin, a midline closure is performed after excising the remaining preputial flap carefully preserving the blood supply to the flap used for the urethroplasty.

**Figure 10:**
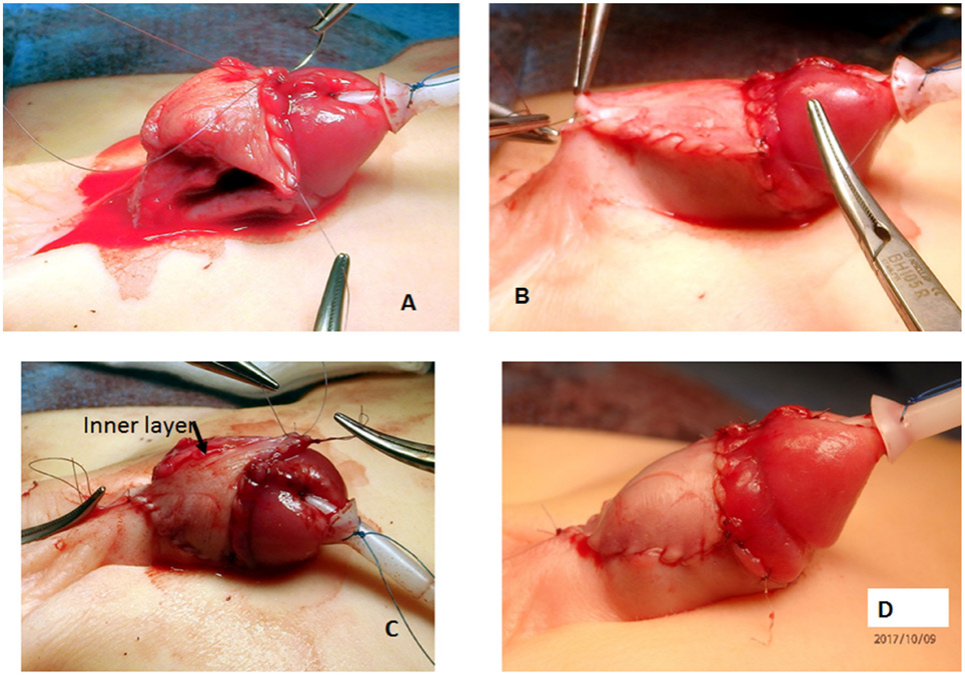
**(A)** The new free edge of the total preputial flap (TPF) is sutured to the ventral coronal skin. **(B)** The other edge is sutured to the side of the penile shaft skin, and finally **(C)** the excess skin of the TPF (including the inner layer) is excised. **(D)** Final appearance.

The dorsal shaft skin is sutured to the corona (Figure 9). Usually, the penile shaft skin is insufficient, and part of the TPF is used to cover the ventral defect (Figure 10). The remaining outer prepuce is usually sufficient, and the inner prepuce is discarded. If correction of a bifid scrotum or penoscrotal transposition is required, it is done at the time of the hypospadias repair.

A lightly compressive dressing is applied. The stent is left in place for 7 days.

**Figure 11:**
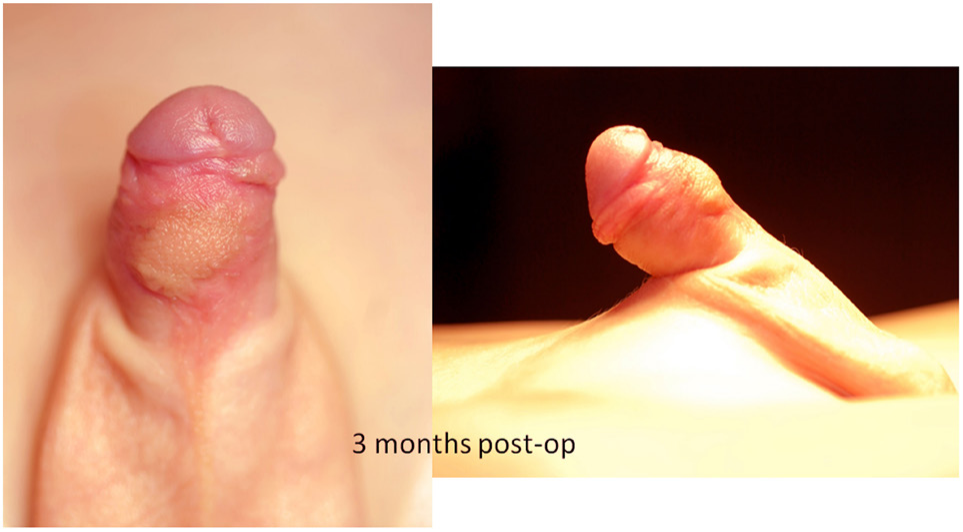
Appearance of the penis 3 months after the repair.

Follow-up visits take place 3 months (Figure 11) postoperatively, when the child is toilet trained and can perform an uroflow study and in the absence of symptoms, at the onset of puberty.

## Results

At institution A, 43 children met the inclusion criteria (17.4% of 248 patients operated for hypospadias in the same period of time). At institution B, the records of six patients were located and reviewed. The results were pooled together and analyzed as a single cohort since there were no significant differences in the patient population or results. The severity of the hypospadias according to the meatal location was (1) glanular, coronal, or distal shaft with a hypoplastic penile urethra and marked chordee in 10 patients; (2) midshaft in 21; (3), penoscrotal in 16; and (4) scrotal or perineal in 2. All patients had penile curvature greater than 20° after degloving the penile skin. Three patients had chromosomal abnormalities: one Klinefelter, one XO, XY asymmetrical gonadal dysgenesis, and one Smith–Lemli–Opitz syndrome.

The mean age at the time of surgery was 21 months (11–110), and the mean duration of follow-up was 23.4 months (1–79). Two patients live in another country and had only the initial postoperative visit. In 48 cases, the plate could be preserved but in one it was divided and an onlay-tube-onlay technique was used (20). In five cases, the onlay flap based on the outer layer of the one preputial flap was used and the ventral skin could be directly approximated. In all other cases, a double preputial flap was used both as an onlay and to cover the ventral penis, as illustrated in Figure 10. The penile curvature was corrected with chordectomy alone in 10 patients, 38 required a dorsal plication of the tunica albuginea, and 1 required an additional ventral dermal graft and 1 division of the urethral plate.

In 38 patients (77.5%), the initial was successful and no further operations were needed. One child with gonadal dysgenesis had a repeat dorsal plication of the tunica albuginea at the time of repair of a glans dehiscence because of residual curvature. There were seven urethrocutaneous fistulas, two dehiscence of the glans approximation, and one child had both a fistula and dehiscence of the glans. One child developed a postoperative wound infection (multi-resistant *Escherichia coli*) and partial necrosis of the flap and is awaiting a reoperation. Four of the seven fistulas were closed with one procedure, one recurred. In three children, the parents chose to delay the fistula repair until later. Three patients with glans dehiscence had successful approximation. We observed no diverticula formation in this series.

**Figure 12:**
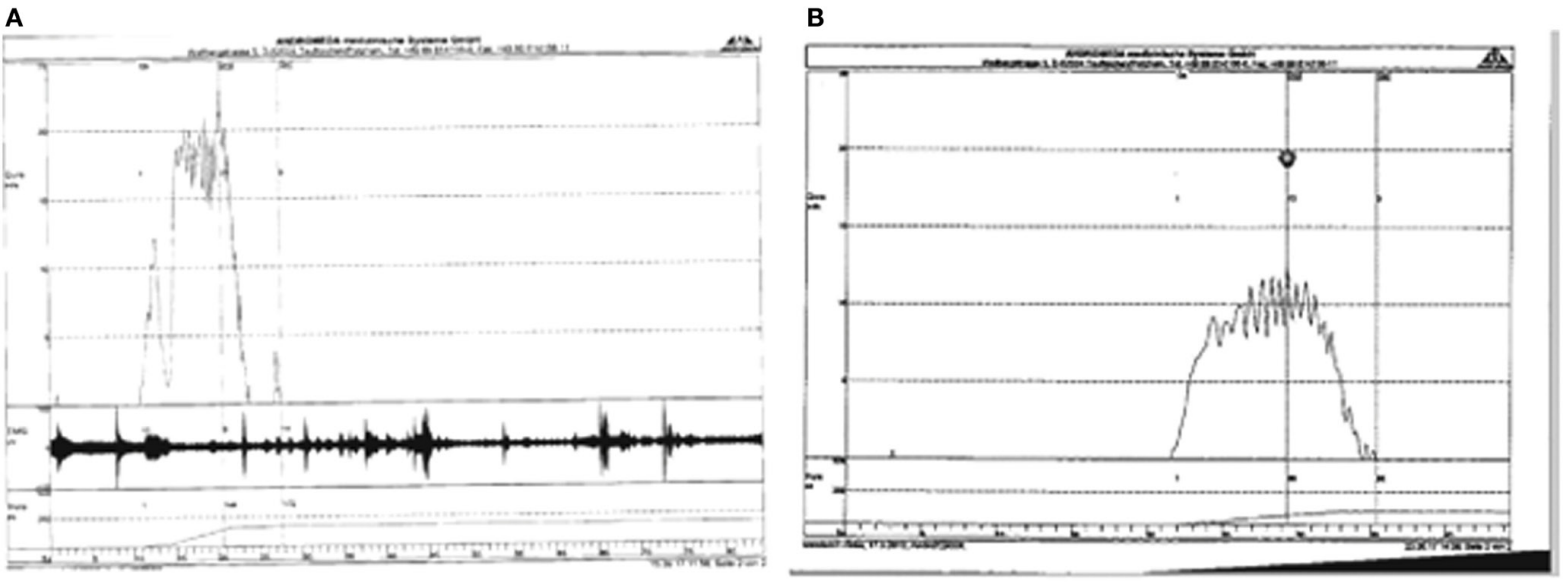
**(A)** Uroflow in child with midshaft hypospadias. Double preputial onlay, 6 years postoperatively. **(B)** Uroflow in child with proximal shaft hypospadias, operated at age 13 months, 3 years postoperatively.

Thirteen patients (26%) were old enough at the time of the last follow-up to obtain an uroflow study. In 11 patients, the flow pattern was bell-shaped (Figure 12), but in 2 the voided volume was too small to be evaluable.

Five patients (10.2%) had preoperative testosterone stimulation to increase the size of the glans if it was less than 14 mm. In this group, there was one dehiscence of the glans and no other complications. Four patients received parenteral testosterone (25 mg intramuscularly) 6 weeks prior to the operation and in one topical testosterone cream was applied. All responded with increased glans size.

## Discussion

The results obtained in this series of patients are comparable to previous reports using the onlay urethroplasty (9). In Barroso et al.’s series also 75% of the children required only one operation for complete resolution of the problem. Using preputial flap urethroplasty with a somewhat different technique, others have reported durable results at a mean follow-up of 14 years in 30 patients (10).

Several points regarding the technique used in this series are worth a comment. We were able to correct all these patients and achieve a straight penis without dividing the urethral plate which allowed the successful use of the onlay technique. Mollard and Castagnola reported 92 cases of proximal hypospadias repaired with preservation of the plate (17, 21), others have stated that division of the urethral plate is necessary in only about 10% of the cases (22, 23). We reserve a planned staged repair only when the glans is less than 14 mm in width even after preoperative testosterone stimulation. In such cases, the use of a free preputial skin graft in the ventral glans facilitates the subsequent urethroplasty. We also use a staged repair for some previously failed operations.

The technique used for construction of the flap is unique in our technique and differs significantly from the typical technique reported to create onlay preputial flaps. Classically, the onlay has been constructed with the inner layer of the prepuce (24) leaving the outer layer, which may be needed to cover the penile shaft (usually as rotational Byars flaps (25)) with poor blood supply (26). In contrast, the use of the TPF (16) ensures that both the skin used for the onlay and the skin use for ventral coverage preserve excellent blood supply. The ventral transposition using a buttonhole at the base of the pedicle prevents axial rotation of the penis, an undesirable outcome that can be seen when the pedicle is brought along a side of the shaft (27). Another difference is the use of the outer layer of the prepuce for the urethroplasty as described by Standoli (11). We attribute the absence of diverticula in this series to the avoidance of the use of the inner prepuce.

In this series, 26% of the patients were able to have an uroflow study which had a bell pattern the majority confirming the absence of urethral stenosis (28).

In our series, the correction of the ventral curvature by removing all paraurethral fibrous tissues and performing a Heineke–Mikulicz plasty in the dorsal midline of the tunica albuginea was satisfactory in 48 of 49 children. One child required a repeat plication at the time of a re-reconstruction of the glans. Our approach to the correction of the curvature with preservation of the urethral plate differed from that proposed by Mollard and Castagnola (17). These authors routinely elevated the urethral plate and only performed a dorsal plication if the curvature persisted. In contrast, we performed the plication first, and when this maneuver was insufficient we elevated the plate and mobilized the urethra proximally. Depending on the degree of correction obtained, if required, we made a transverse incision in the ventral aspect of the tunica albuginea and covered the defect with a graft of tunica vaginalis or dermis. Of course, it is impossible to assess the success of the repair of the ventral curvature unless the patients are followed until the onset of sexual activity.

Although the numbers are too small to draw conclusions, in this series the use of preoperative testosterone stimulation which enabled us to do the procedure did not increase the complication rate.

We were able to correct the malformation in 77.5% of the children with one operation. Others have argued that a staged operation has a lower complication rate. For example, following the principles described by Bracka et al. (13), Pfistermüller et al. had an impressively low reoperation rate of 6.25% after the second stage of a staged repair in 208 patients (12). Nevertheless, their group of patients required 429 procedures under general anesthesia until resolution of the problem or 2.07 procedures per child. In another series of repairs using a modified Bracka technique (29) of 43 cases, 77% were solved with 2 operations (total number 86) and 10 other patients required 1 or 2 additional procedures for a total number of procedures of 2.23 per child. With the technique described here, fewer than 1.2 procedures per patient were required.

With increasing concerns about potential adverse effects of repeated general anesthesia (30) as well as with concerns about cost of care and efficient utilization of hospital beds, we postulate that the technique described here is more efficient than a planned staged procedure (31).

The limitations of the study are clear and include the following: retrospective nature, short follow-up, small number of patients, lack of objective assessment of cosmesis, or correction of chordee. Unfortunately, these shortcomings are shared by the majority of reports on hypospadias repair. Nevertheless, one of the authors has used this technique for the last 25 years for the majority of primary repair of proximal hypospadias (15).

Another limitation of the interpretation of the results is that two patients were lost to follow-up and that five patients had less than 6-month follow-up. If we eliminate these 6 patients altogether from the series, 32 of 43 (74%) were complication free.

We conclude that the repair of mid- and proximal hypospadias with preservation of the plate can be done in the majority of cases. An onlay urethroplasty using the outer layer of a TPF, in most cases also for ventral skin coverage, yields results comparable to those of staged techniques and results in fewer procedures under anesthesia after a mean follow-up of 23.4 months.

## Ethics Statement

Ethical approval was obtained from the Ethical Commitee of the Medizinisches Hochschule Hannover (MHH) to review all records of patients undergoing hypospadias operations at the Auf der Bult Jugend-und Kinderkrankenhaus. No ethical approval required for a retrospective review at Medizinsuniversität Berlin Charité Campus Virchow.

## Author Contributions

The data were collected and analyzed by the three authors. Operations were performed by AL and BL. RG wrote the manuscript and prepared the figures.

## Conflict of Interest Statement

The authors declare that the research was conducted in the absence of any commercial or financial relationships that could be construed as a potential conflict of interest.
